# A double-blind placebo controlled trial into the impacts of HMB supplementation and exercise on free-living muscle protein synthesis, muscle mass and function, in older adults

**DOI:** 10.1016/j.clnu.2018.09.025

**Published:** 2019-10

**Authors:** U.S.U. Din, M.S. Brook, A. Selby, J. Quinlan, C. Boereboom, H. Abdullah, M. Franchi, M.V. Narici, B.E. Phillips, J.W. Williams, J.A. Rathmacher, D.J. Wilkinson, P.J. Atherton, K. Smith

**Affiliations:** a MRC-ARUK Centre for Musculoskeletal Ageing Research, Clinical, Metabolic and Molecular Physiology, University of Nottingham, Royal Derby Hospital Centre, Derby, UK; b Nottingham NIHR BRC, UK; c Metabolic Technologies, Inc, Iowa State University Research Park, 2711 S. Loop Drive, Ste 4400, Ames, IA, 50010, USA

**Keywords:** Skeletal muscle, HMB, Stable isotopes, Exercise, D_2_O, D_2_O, Deuterium oxide, MVC, Maximal voluntary contraction, RET, Resistance exercise training, 1-RM, One repetition maximum, TC/EA, High temperature conversion elemental analyser, GC, Gas chromatography, IRMS, Isotope ratio mass spectrometer, MPS, Muscle protein synthesis, USG, Ultrasonography, DXA, Dual-energy X-ray absorptiometry, VL, Vastus lateralis, MCME, *N –*Methoxycarbonyl methyl ester, APE, Atom percent excess, RPE- Rating of perceived exertion, PLA- Placebo, HMB, β-hydroxy-β-methyl-butyrate, HMB-FA, Free Acid β-hydroxy-β-methyl-butyrate, UT, Untrained leg, RE, Resistance exercise

## Abstract

Age-related sarcopenia and dynapenia are associated with frailty and metabolic diseases. Resistance exercise training (RET) adjuvant to evidence-based nutritional intervention(s) have been shown as mitigating strategies. Given that β-hydroxy-β-methyl-butyrate (HMB) supplementation during RET improves lean body mass in younger humans, and that we have shown that HMB acutely stimulates muscle protein synthesis (MPS) and inhibits breakdown; we hypothesized that chronic supplementation of HMB free acid (HMB-FA) would enhance MPS and muscle mass/function in response to RET in older people.

We recruited 16 healthy older men (Placebo (PLA): 68.5 ± 1.0 y, HMB-FA: 67.8 ± 1.15 y) for a randomised double-blind-placebo controlled trial (HMB-FA 3 × 1 g/day vs. PLA) involving a 6-week unilateral progressive RET regime (6 × 8 repetitions, 75% 1-RM, 3 · wk^−1^). Deuterium oxide (D_2_O) dosing was performed over the first two weeks (0–2 wk) and last two weeks (4–6 wk) with bilateral vastus lateralis (VL) biopsies at 0–2 and 4–6 wk (each time 75 ± 2 min after a single bout of resistance exercise (RE)) for quantification of early and later MPS responses and post-RE myogenic gene expression. Thigh lean mass (TLM) was measured by DXA, VL thickness and architecture (fibre length and pennation angle) by ultrasound at 0/3/6 wk, and strength by knee extensor 1-RM testing and MVC by isokinetic dynamometry (approx. every 10 days).

RET induced strength increases (1-RM) in the exercised leg of both groups (398 ± 22N to 499 ± 30N HMB-FA vs. 396 ± 29N to 510 ± 43N PLA (both P < 0.05)). In addition, maximal voluntary contraction (MVC) also increased (179 ± 12 Nm to 203 ± 12 Nm HMB-FA vs. 185 ± 10 Nm to 217 ± 11 Nm PLA (both P < 0.05); with no group differences. VL muscle thickness increased significantly in the exercised leg in both groups, with no group differences. TLM (by DXA) rose to significance only in the HMB-FA group (by 5.8%–5734 ± 245 g p = 0.015 vs. 3.0% to 5644 ± 323 g P = 0.06 in PLA). MPS remained unchanged in the untrained legs (UT) 0–2 weeks being 1.06 ± 0.08%.d^−1^ (HMB-FA) and 1.14 ± 0.09%.d^−1^ (PLA), the trained legs (T) exhibited increased MPS in the HMB-FA group only at 0–2-weeks (1.39 ± 0.10%.d^−1^, P < 0.05) compared with UT: but was not different at 4–6-weeks: 1.26 ± 0.05%.d^−1^. However, there were no significant differences in MPS between the HMB-FA and PLA groups at any given time point and no significant treatment interaction observed. We also observed significant inductions of c-Myc gene expression following each acute RE bout, with no group differences. Further, there were no changes in any other muscle atrophy/hypertrophy or myogenic transcription factor genes we measured.

RET with adjuvant HMB-FA supplements in free-living healthy older men did not enhance muscle strength or mass greater than that of RET alone (PLA). That said, only HMB-FA increased TLM, supported by early increases in chronic MPS. As such, chronic HMB-FA supplementation may result in long term benefits in older males, however longer and larger studies may be needed to fully determine the potential effects of HMB-FA supplementation; translating to any functional benefit.

## 1 Introduction

Age-related loss of skeletal muscle mass and function is a well-established hallmark of sarcopenia [1]. In spite of the fact that clinical diagnostics for sarcopenia are not yet available, there have been a number of iterations of defining criteria, including those based on low muscle mass for age [2], or more recently, composites of low muscle mass and function [3]. However, despite some progress in defining sarcopenia, there remains no widely accepted pharmacological therapy to treat it. This has led to a host of research aimed at testing adjuvant nutritional and exercise interventions that may be able to facilitate skeletal muscle maintenance in older age.

Resistance exercise training (RET) remains the most potent anabolic stimulus in relation to skeletal muscle tissue, through the induction of cellular hypertrophy [4]. Muscle growth associated with RET is also accompanied by functional improvements [5], [6] such as increases in isokinetic/isometric strength and in power, making this an attractive intervention for maintenance of muscle function in older age. That being said, gains in muscle mass are blunted in older age when compared to those in younger people [6], [7] and meta-analyses suggest that gains in lean mass with long-term RET equate to just ∼1 kg in older people [8]. Thus, novel strategies to bolster anabolic responses to RET in older age are needed.

In order for skeletal muscle hypertrophy to occur, sufficient exogenous amino acids (derived from dietary protein sources) are required. This is perhaps why protein supplements have been shown to incrementally enhance RET-induced gains in muscle mass in both younger and older adults alike [9]; though whether this “effect” is a facet only impacting those with lower habitual intakes remains unknown. Beyond protein supplementation, the post-transamination product of leucine/alpha-ketoisocaproic acid, Beta-Hydroxy Beta-Methylbutyrate (HMB) has received considerable attention as an ergogenic nutraceutical (i.e. a performance enhancing nutritional supplement) either alone or in tandem with RET [10]. HMB has many purported mechanisms of action (summarised in the following review [11]) but in terms of promoting muscle hypertrophy, has been shown to stimulate muscle protein synthesis (MPS) and suppress muscle protein breakdown (MPB) acutely in younger males [12]. There have been numerous studies investigating the efficacy of HMB supplements alongside RET. For example, chronic HMB supplementation has been shown to increase muscle mass and strength in younger individuals undergoing RET [13] and also in older women undertaking a twice weekly exercise programme [14]. Similarly, long term (∼1y) supplementation with a HMB/arginine/lysine mixture improved muscle mass and strength in non-vitamin D deficient older adults [15]. This is further supported by a two-phase study whereby calcium HMB supplements alone improved muscle mass gains compared to a placebo, although there was no additive impact with RET, [16]. Finally, despite varying reports of efficacy [11], a meta-analysis concluded that HMB supplements contribute to the preservation of muscle mass in old age [17].

In the present study, we aimed to determine the impact of HMB supplementation in older males undertaking unilateral RET, and to define the underlying mechanisms of any effects of HMB using newly developed techniques that permit the long-term measures of MPS via deuterium oxide (D_2_O) [5], [6], [18]. Furthermore, we adopted the use of the free acid (HMB-FA) rather than the calcium form (Ca-HMB) of HMB, since greater bio-availability of HMB-FA has been shown in humans [19], [20]. We hypothesised HMB-FA supplementation would enhance muscle mass gains with/without RET by enhancing MPS.

## 2 Materials and methods

### 2.1 Subject characteristics and ethics

Sixteen healthy older males were recruited and randomly assigned (8 per group) to either HMB-FA (Age 67.8 ± 1.1 y, BMI 28 ± 1 kg m^−2^) or the placebo (Age 68.5 ± 1.6 y, BMI 27 ± 1 kg m^−2^) group; see Table 1 for subject characteristics. Sample size was powered based on the primary end point of chronic MPS. A sample size of 8 in each group was determined to have a >80% power to detect a mean difference in MPS of 25% assuming that the pooled SD of chronic MPS measures is 0.23 (based on previous data from our lab [5], [6], [18], and a Cohen's d estimation of effect size of 1.55 using a two-group unpaired t-test with a 0.05 two-sided significance. All power calculations were performed using the pwr R package (https://cran.rproject.org/web/packages/pwr/index.html). All volunteers were screened by medical questionnaire, physical examination, and resting electrocardiogram, with exclusions for metabolic, respiratory, and cardiovascular disorders or any other symptoms of ill health. Subjects had normal blood chemistry, were normotensive and were not prescribed any medications; all subjects performed activities of daily living and recreation but did not undertake in any RET other than that described in the study and had not recently participated in any RET. All subjects provided their written, informed consent to participate after all procedures and risks (in relation to muscle biopsies, blood sampling, etc.) were explained. This study was approved by The University of Nottingham Ethics Committee and complied with the Declaration of Helsinki and registered at https://clinicaltrials.gov/(NCT02505438). All analysts and those involved in the conduct of the study were blinded throughout as to which treatment group the samples or volunteers belonged.

**Table 1:** Shows baseline demographics for both groups, there were no significant differences between the groups.

	Placebo	Treatment
Age (y)	68.5 ± 1.0	67.8 ± 1.1
Height (m)	1.74 ± 0.02	1.76 ± 0.02
Weight (kg)	83 ± 2.9	86 ± 4.1
BMI (kg.m^−2^)	27 ± 0.9	28 ± 1.2
ASM (kg)	24.8 ± 1.1	25.3 ± 1.3
ASMI (ASM kg.m^−2^)	7.8 ± 0.3	8.5 ± 0.4
Thigh Lean Mass (kg)	5.5 ± 0.3	5.6 ± 0.3

### 2.2 Conduct of the study

**Fig. 1:**
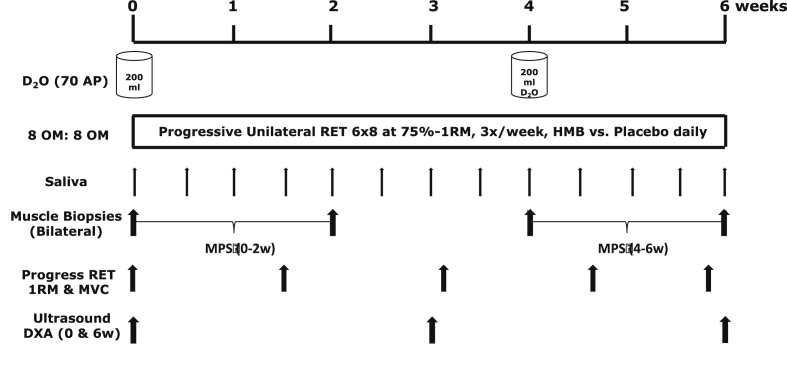
Study protocol.

This study involved a bilateral protocol whereby one leg was used as an untrained (UT) internal control with the other leg acting as the trained (T) leg, with the dominant leg assigned as T. Following inclusion to the study, subjects were studied over a 6-week period. On the first day of study, subjects arrived at the laboratory at 0830 h following an overnight fast. Following assessment of *Vastus Lateralis* (VL) skeletal muscle architecture by ultrasound (Mylab 70; Esaote Biomedical) and body composition by dual-energy X-ray absorptiometry (DXA; Lunar Prodigy II, GE Medical Systems, Little Chalfont, Buckinghamshire, UK), subjects completed the 1st session of RET consisting of unilateral knee-extension exercise (i.e. 6 × 8 reps at 75% 1-RM). Bilateral biopsies were taken from VL muscle 75 ± 2 min after unilateral exercise under sterile conditions, using the conchotome biopsy technique [21] with 1% lidocaine (B. Braun Melsungen) as local anaesthetic. Muscle was rapidly dissected free of fat and connective tissue, washed in ice-cold PBS, blotted to remove excess water, frozen rapidly in liquid N_2,_ then stored at −80 °C until further analysis. Immediately post-RET, subjects provided a saliva sample (collected in sterile plastic tubes) then consumed a 200 ml bolus of D_2_O (70 atom%; Sigma–Aldrich, Poole, UK); with the aim to label the body water pool to ∼0.2% atom percent excess (APE); this dosing protocol was repeated at week 4 thereby providing two distinct periods of measurement of MPS. In addition, venous blood samples were collected into lithium heparin coated tubes, immediately cold centrifuged at 1750 g, with plasma fractions aliquoted and frozen at −80 °C until analysis. Thereafter, subjects returned to the lab 3 · wk^−1^ to undertake supervised unilateral RET with 1-RM assessments of the T leg every ∼10 d to ensure progressive intensity. Further bilateral muscle biopsies (∼75 min after RET in order to investigate the temporal nature of acute anabolic signalling responses to progressive RET), ultrasound measures of muscle architecture and venous blood samples were taken at 0, 2, 4 and 6-weeks and muscle thickness and lean thigh mass by DXA at 0, 3 and 6-weeks. For the temporal monitoring of body water enrichment, each participant provided a saliva sample on RET-visits at least 30 min after their last meal or drink, with extra samples taken ∼3 h after bolus top-ups to ensure body water enrichment was accurately determined over time. These were collected in sterile plastic tubes and immediately cold centrifuged at 16,000 g to pellet any debris; they were then aliquoted into 2-ml glass vials and frozen at −20 °C until analysis. A detailed representative schematic of the study protocol is depicted in Fig. 1.

### 2.3 Exercise training protocol

The 1 repetition maximum (1-RM) was determined for the dominant leg prior to the study beginning. Supervised unilateral RET (leg extension of the dominant leg, 6 sets of 8 reps at 75% 1-RM, three times weekly) was performed over 6 weeks. RET was progressive and the 1-RM, and MVC was assessed every 10 days. This intensity of exercise has previously been shown to maximize MPS responses acutely in older males [22].

### 2.4 Treatment supplementation

All subjects were randomly assigned to either placebo or HMB-FA (3.0 g/d; Metabolic Technologies, Ames, IA); volunteers were asked to consume the supplement daily at breakfast. Each HMB-FA sachet consisted of 1 g of β-hydroxy-β-methylbutyric free acid (BetaTOR^®^), Litesse polydextrose, reverse osmosis water, debittering agent, orange flavour, stevia extract, potassium carbonate and potassium sorbate; each sachet of placebo consisted of a similar amount of Litesse polydextrose, reverse osmosis water, corn syrup, debittering agent, orange flavouring, stevia extract, citric acid, potassium sorbate, and xanthin gum powder. Supplements were obtained in identical plain packaging, coded by the manufacturer, all investigators remained blinded until study and analyses were completed. Volunteers were first assigned to a lettered group, by a clinical measurement technician (AG, see acknowledgments), based on random allocation using a sealed envelope; then received the supplements weekly (3 sachets per day), and a log of returned sachets was kept to inform upon compliance, which was excellent in both groups (100% PLA vs 99% HMB). Blood samples were collected weekly immediately prior to the RET for the measurement of plasma HMB concentrations.

### 2.5 Muscle functional tests

Measures of maximal voluntary contraction (MVC) and one repetition maximum (1-RM) were assessed. MVC was measured with isometric contractions conducted in a sitting position using an isokinetic dynamometer (Isocom; Isokinetic Technologies, Eurokinetics) over a range of four knee joint angles (60°, 70°, 80° & 90°), with full extension corresponding to 0°. Subjects were seated in the dynamometer chair and secured into position using a chest strap. Contractions lasted 4 s, with a 30 s rest period between contractions and 90 s between knee joint angles. Unilateral leg extension 1-RM was assessed on the dominant leg (Technogym, Gambettola, Italy). Following explanation of the procedure, subjects performed a light warm up to avoid injury and ensure familiarity whilst avoiding fatigue; 1-RM was then achieved (20 on Borg's RPE scale) in as few repetitions as possible with a maximum of 5 repetitions. The first repetition was estimated at 50% 1-RM and then increased until subjects could not complete controlled contraction throughout the range of motion, with 3 min rest in-between attempts.

### 2.6 Muscle architecture, DXA derived mass

Muscle architecture was measure as described [18]. Briefly, the architectural parameter of muscle thickness (MT), was quantified from ultrasound scans using Image J 1.42q (National Institutes of Health, Bethesda, MD). The MT was measured as the distance between the superficial and deep aponeuroses (Franchi et al., 2014). DXA derived quadriceps muscle mass was determined from the lowest point of the ischium to the knee space.

### 2.7 Body water and protein bound alanine muscle fraction enrichment

Body water and muscle protein enrichment was measured as previously described [18]. Saliva was heated in a vial at 100 °C, then cooled rapidly on ice and the condensate transferred to a clean vial ready for analysis. Deuterium enrichment was measured on a high-temperature conversion elemental analyser connected to an isotope ratio mass spectrometer (TC/EA-IRMS Thermo Finnigan, Hemel Hempstead, UK). Myofibrillar proteins were extracted as previously described; briefly, the myofibrillar fraction was isolated by centrifugation from the sarcoplasmic protein solubilized in 0.3M NaOH and separated from the insoluble collagen by centrifugation. The protein was then precipitated, acid hydrolysed, and the free amino acids were purified and derivatised as their n-methoxycarbonyl methyl esters (MCME) (Wilkinson 2014). Incorporation of deuterium into protein bound alanine was determined by gas chromatography-pyrolysis-isotope ratio mass spectrometry (GC-pyrolysis-IRMS, Delta V Advantage; Thermo Finnigan, Hemel Hempstead, UK) alongside a standard curve of known dl-Alanine-2,3,3,3-d4 enrichment to validate measurement accuracy.

### 2.8 Calculation of FSR

Myofibrillar MPS was calculated from the incorporation of deuterium-labelled alanine into protein, using the enrichment of body water (corrected for the mean number of deuterium moieties incorporated per alanine, 3.7, and the dilution from the total number of hydrogens in the derivative, 11) as the surrogate precursor labelling between subsequent biopsies. The equation used was:

$$FSR=-Ln\left(\frac{1-\left[\frac{\left(APEala\right)}{\left(APEp\right)}\right]}{t}\right)$$

where *APE*_*Ala*_ = deuterium enrichment of protein-bound alanine, *APE*_*P*_ = Mean precursor enrichment over the time period, and *t* is the time between biopsies.

### 2.9 RNA extraction

Muscle biopsies were taken from both rest and exercise legs of the subjects at 0, 2, 4 and 6-week time points. Muscle tissue (5–10 mg) was chipped, homogenized in TRIzol Reagent and lysed (TissueLyserII, QIAGEN). The RNA was extracted in the upper aqueous phase, then precipitated with isopropanol, washed, taken up in RNase free water, then the purity and quantity were assessed (NanoDrop Lite, Thermo Scientific). A ratio of 1.7–2.0 was accepted as ‘pure’ for RNA. An aliquot of RNA was then reverse transcribed (High Capacity cDNA Reverse Transcription Kit; Life Technologies) according to the manufacturer's instructions and stored at −20 °C until qRT-PCR was performed.

### 2.10 Measurement of gene expression by qRT PCR

#### 2.10.1 Primer design

Real-time RT-PCR exon specific primers were designed for myostatin, MyoD, myogenin, human muscle atrophy F-box (MAFbx/Atrogin-1), human calpain, Ubiquitin, cMyc, Muscle RING-finger protein-1 (MURF1), Myogenic factor 5 (MYF5), REDD1 (regulated in development and DNA damage responses 1), eIF3f (eukaryotic translational initiation factor 3, subunit F) and Peptidylprolyl isomerase A (PPIA3) gene using Primer express software. Finally, all sequences were submitted to the Primer blast software, available on the National Centre for Biotechnology Information website (www.ncbi.nlm.nih.gov), in order to evaluate gene specificity. All mRNA-specific primers were validated before use.

All measures were carried out in triplicate, and the values of amplification were normalized to the “housekeeping gene” Peptidylprolyl isomerase A (PPIA3), in each biopsy and compared to the control leg on Day 0, since its expression remains unaltered with exercise. Sybr-Green real-time RT-PCR analyses were carried out on the iQ5 real-time PCR detection system (Bio-Rad). A melt curve was run at the end of each qRT-PCR assay to validate product specificity. For each qRT-PCR experiment, a standard curve was generated for each target gene. The relative gene expression were calculated using the 2^−Δ*CT*^ method [23]. This method generates a value in arbitrary units (AU) of the gene of interest (GOI) expression normalized to the housekeeping gene (HKG) expression (ΔCT = C_TGOI_ - C_THKG_).

### 2.11 Statistical analyses

Descriptive statistics were produced for all data sets to check for normal distribution (accepted if P > 0.05) using a Kolmogorov–Smirnov test. Data from all subjects (n = 8 per group) are presented as means ± SEM. Myofibrillar protein synthesis rates and all other data sets were analysed by repeated measures two-way ANOVA with a Bonferroni correction using GraphPad Prism (La Jolla, CA) Software Version 5. The α level of significance was set at P < 0.05.

## 3 Results

### 3.1 1-RM and MVC in rest and exercise legs (Fig. 2)

Over the 6-week period, RET increased 1-RM linearly in the trained leg in both groups, (baseline 396 ± 29N to 510 ± 44N, +29% (PLA) and 398 ± 22N to 499 ± 31N, +25% (HMB-FA), both p < 0.01 compared to baseline, with no difference between the groups). Similarly, MVC increased progressively in the trained leg of both groups (baseline 185 ± 10 Nm vs. 217 ± 11 Nm, +18% (PLA) and 179 ± 12 Nm to 203 ± 12 Nm, +16% (HMB-FA) at 6 weeks, both p < 0.05 vs baseline, with no differences between groups). Both 1-RM and MVC were unchanged in the control legs of either group.

**Fig. 2:**
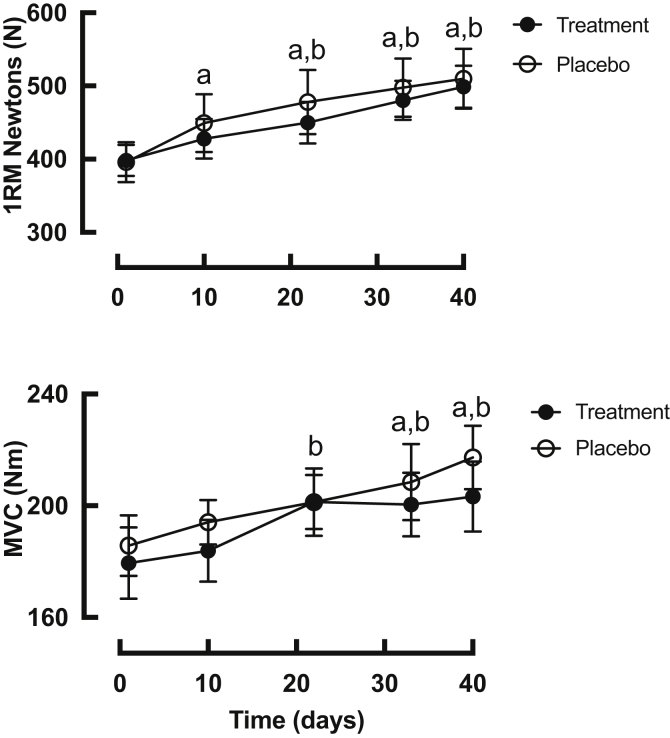
Time course of changes in 1-RM strength (upper) and MVC (lower) of trained leg in both treatment (HMB-FA) and placebo groups. a – p < 0.05 significantly different from baseline in placebo group. b-p < 0.05 significantly different from baseline in treatment group. There was no significant differences between the groups at any time-point.

### 3.2 Muscle mass and architecture in rest and exercise legs (Fig. 3)

**Fig. 3:**
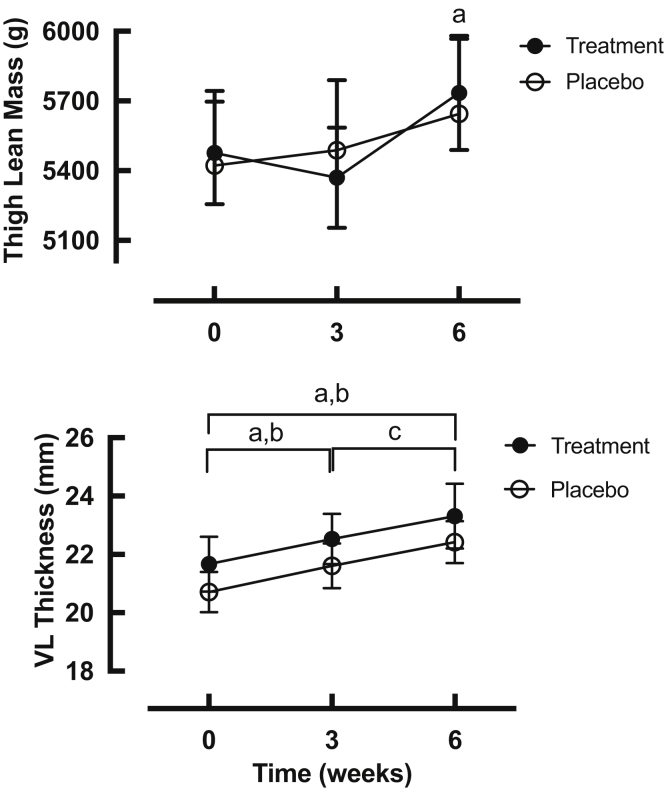
Temporal changes in thigh lean mass (upper) and VL thickness (lower), in the exercised legs over 6-week training, in both treatment (HMB-FA) and placebo groups. a – p < 0.05 significantly different from baseline in treatment group, b – p < 0.05 significantly different from baseline in placebo group, c-p < 0.05 significantly different from previous time-point for placebo group. There were no significant differences between the groups at any time-point.

Thigh lean mass assessed by DXA was not significantly different between the groups at the start of the study (i.e. baseline 5423 ± 321 g PLA vs 5476 ± 221 g HMB-FA), with only the HMB-FA group significantly increasing lean muscle mass in the trained leg (Fig 3, 5734 ± 245 g at 6 weeks HMB-FA, P < 0.05 vs 5644 ± 323 g PLA, P = 0.06). Thigh fat free mass was unchanged in the control legs of both groups (5338 ± 300 g to 5311 ± 274 g PLA, and 5399 ± 262 g to 5470 ± 237 g HMB-FA). There were no differences between the groups at any time. Measures of VL architecture (Fig. 3) revealed significant increases in MT over 6 weeks in exercise legs of both groups (20.7 ± 0.6 mm to 22.4 ± 0.7 mm PLA, and 21.7 ± 0.9 mm to 23.3 ± 1.1 mm HMB-FA, both P < 0.01. Muscle thickness was unchanged over the 6 week period in the control leg in both placebo and HMB-FA groups. Pennation angle increased significantly in the exercised leg in both groups by 6 weeks (14.1 ± 1.8° to 15.2 ± 1.5° PLA, and 13.7 ± 1.5° to 14.7 ± 1.8° HMB-FA P < 0.05) and was unchanged in the control leg over the same period. Fibre length (Lf) was increased significantly in the exercised leg in both groups at 6 weeks (79.7 ± 2.8 cm to 83.1 ± 3.3 cm PLA, and 81.0 ± 6.1 cm to 84.0 ± 6.4 cm HMB-FA, both P < 0.001, with no difference between the groups).

### 3.3 Plasma HMB concentrations

**Fig. 4:**
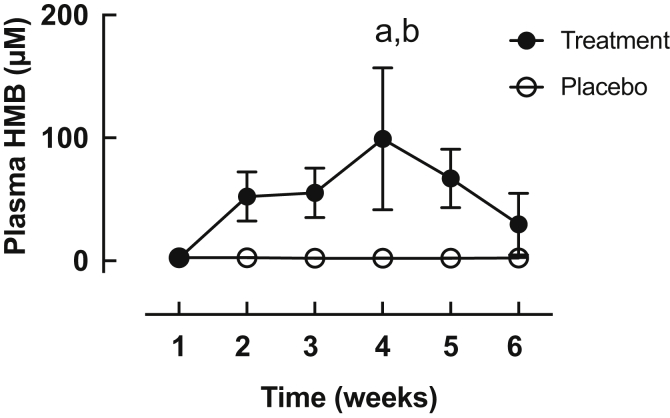
Plasma HMB concentration in the treatment (HMB-FA) and placebo groups following oral consumption of treatment or placebo. Statistical significance (p < 0.05) a = different from respective basal, b = different between groups at equivalent time-point.

Plasma HMB concentration was significantly increased throughout only in subjects in the treatment group (Fig. 4), whereas plasma HMB remained constant (i.e. at baseline, ∼2.4 ± 0.2 μM) throughout the study in the placebo group.

### 3.4 D_2_O-derived muscle protein synthesis (MPS) in rest and exercised legs of placebo and treatment group (Fig. 5)

Over 0–2 weeks there was no significant treatment interaction between the PLA and HMB-FA groups, however significant increases in MPS were displayed from 0 to 2 weeks in the exercise leg with HMB-FA supplementation only (1.06 ± 0.08%.d^−1^ Rest vs 1.39 ± 0.10%.d^−1^ Exercise, P < 0.05, thereafter returning to 1.26 ± 0.05%.d^−1^ at 4–6 weeks). MPS rose slightly but not significantly in the exercised leg of the placebo group (1.14 ± 0.09%.d^−1^ Rest vs 1.36 ± 0.10%.d^−1^ Exercise, thereafter returning to 1.23 ± 0.05%.d^−1^ at 4–6 weeks). MPS was not different between legs nor treatments over 4–6 weeks. In addition, we did not find any significant differences in MPS between placebo or treatment at any time-point.

**Fig. 5:**
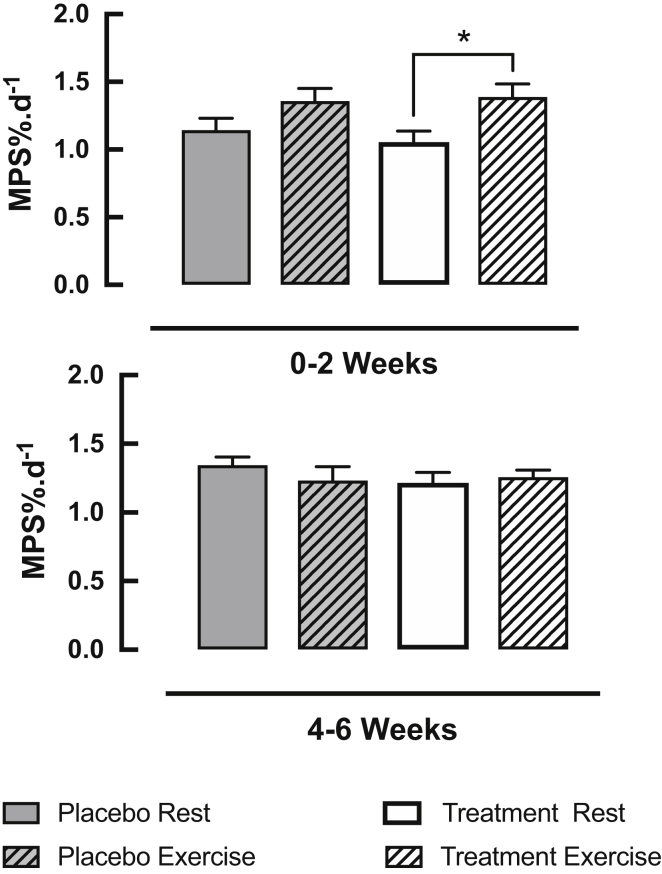
Depicts time course of changes in muscle protein synthesis (MPS) over 0–2 and 4–6 weeks in both rest and exercised legs of placebo and treatment (HMB-FA) groups. *P < 0.05 statistically significant in trained leg of treatment group as compared to rest leg.

### 3.5 Expression of genes involved in muscle hypertrophy/atrophy and myogenesis

Of all the genes measured (see Table 2), only cMyc mRNA expression changed significantly (P < 0.05) in response to an acute bout of RET. In both the placebo and treatment groups, cMyc mRNA expression levels were elevated in the trained leg at 0, 2 and 4 weeks compared to the untrained leg at each timepoint. However, this response was ameliorated in both groups at week 6. There were no changes in cMyc expression in the rest leg in either group at any time-point and no significant differences between the placebo and treatment groups at any time point.

**Table 2:** Muscle mRNA expression.

	Groups	BASAL		2 week		4 week		6 week	
		Rest	Acute RET	Rest	Acute RET	Rest	Acute RET	Rest	Acute RET
**cMyc**									
5′-GGTAGTGGAAAACCAGCAGCC-3′	PLA	1.0 ± 0.7	3.0 ± 2.0*	1.0 ± 0.6	4.9 ± 0.7*	0.6 ± 0.4	3.0 ± 3.3*	1.2 ± 1.3	2.2 ± 1.0
5′-TCTCCTCCTCGTCGCAGTA-3′	HMB	0.9 ± 0.6	3.0 ± 1.9*	1.9 ± 3.8	3.0 ± 1.9*	0.6 ± 0.3	2.6 ± 1.7*	1.5 ± 2.2	1.7 ± 2.0
**Myostatin**									
5′-GCTGCGCCTGGAAACAGCTC-3′	PLA	1.0 ± 0.3	1.1 ± 0.6	1.7 ± 1.3	1.0 ± 0.4	1.1 ± 0.5	0.8 ± 0.3	1.0 ± 0.6	1.4 ± 0.8
5′-ATCAGTTCCCGGAGTGGAGGC-3′	HMB	1.3 ± 0.7	1.1 ± 0.5	1.6 ± 0.8	0.8 ± 0.3	1.1 ± 0.6	1.0 ± 0.7	0.9 ± 0.5	1.1 ± 0.4
**Myogenin**									
5′-ACCCTACAGATGCCCACAAC-3′	PLA	1.0 ± 0.4	0.7 ± 0.4	1.1 ± 0.9	0.7 ± 0.2	0.4 ± 0.3	0.9 ± 0.8	1.0 ± 0.6	0.9 ± 0.6
5′-GGAAGGCCACAGACACATCT-3′	HMB	0.5 ± 0.2	1.0 ± 0.5	0.5 ± 0.3	0.7 ± 0.4	1.3 ± 0.9	0.5 ± 0.2	1.2 ± 1.5	0.6 ± 0.3
**MyoD**									
5′-CTCCGACGGCATGATGGACTA-3′	PLA	1.0 ± 0.6	0.8 ± 0.4	1.3 ± 0.5	0.8 ± 0.6	0.5 ± 0.4	0.5 ± 0.3	0.6 ± 0.3	0.9 ± 0.5
5′-TGGGCGCCTCGTTGTAGTA-3′	HMB	2.5 ± 1.5	1.9 ± 1.1	3.3 ± 1.3	2.1 ± 1.6	1.1 ± 1.0	1.2 ± 0.7	1.6 ± 0.7	1.3 ± 1.3
**Myf5**									
5′-ATGCCCGAATGTAACAGTCCT-3′	PLA	1.0 ± 0.5	1.0 ± 0.5	0.7 ± 0.3	0.7 ± 0.4	0.3 ± 0.2	0.6 ± 0.4	0.7 ± 0.3	1.0 ± 0.6
5′-GGTTGCTCTGAGGAGGTGAT-3′	HMB	0.9 ± 0.8	0.5 ± 0.5	0.8 ± 0.9	0.7 ± 0.6	0.5 ± 0.3	0.6 ± 0.3	0.6 ± 0.4	0.7 ± 0.4
**eIF3f**									
5′-GTTGGGAACTGTCGACAAAC-3′	PLA	1.0 ± 0.5	0.9 ± 0.3	0.7 ± 0.2	1.0 ± 0.7	0.5 ± 0.3	0.6 ± 0.2	0.6 ± 0.2	0.9 ± 0.5
5′-ATTCCATGTCAACAGCCACT-3′	HMB	0.8 ± 0.4	0.7 ± 0.3	0.7 ± 0.2	0.7 ± 0.5	0.5 ± 0.2	0.6 ± 0.3	0.6 ± 0.1	0.5 ± 0.1
**REDD1**									
5′-AAGACACGGCTTACCTGGA-3′	PLA	1.0 ± 0.6	1.0 ± 0.6	0.8 ± 0.4	0.5 ± 0.2	0.4 ± 0.2	0.6 ± 0.5	0.8 ± 0.4	1.0 ± 0.9
5′-CAGTAGTTCTTTGCCCACCT-3′	HMB	0.9 ± 0.6	0.9 ± 0.6	0.6 ± 0.3	0.7 ± 0.4	0.5 ± 0.3	1.1 ± 0.8	0.8 ± 0.4	0.4 ± 0.1
**MURF1**									
5′-AGTGACCAAGGAGAACAGTCA-3′	PLA	1.0 ± 1.2	2.3 ± 1.6	0.6 ± 0.3	1.5 ± 1.6	0.4 ± 0.3	0.6 ± 0.4	0.5 ± 0.4	0.6 ± 0.4
5′-CACCAGCTTTGTGGACTTGT-3′	HMB	0.4 ± 0.2	0.8 ± 0.4	0.3 ± 0.2	0.7 ± 0.6	0.2 ± 0.1	0.6 ± 0.4	0.4 ± 0.2	0.4 ± 0.2
**MAFbx**									
5′-CCTTCACTGACCTGCCTTT-3′	PLA	1.0 ± 0.5	1.8 ± 1	0.8 ± 0.5	0.7 ± 0.7	0.7 ± 0.9	0.6 ± 0.3	0.4 ± 0.2	0.7 ± 0.4
5′-CCTTCACTGACCTGCCTTT-3′	HMB	0.6 ± 0.4	0.5 ± 0.3	0.4 ± 0.2	0.6 ± 0.5	0.3 ± 0.2	0.5 ± 0.4	0.4 ± 0.2	0.3 ± 0.1
**Ubiquitin**									
5′-GATCTGCCGCAAGTGCTA-3′	PLA	1.0 ± 2.0	1.1 ± 1.6	1.1 ± 2.4	1.1 ± 2.4	0.5 ± 1.2	0.6 ± 0.6	0.4 ± 0.6	0.5 ± 0.8
5′-CAGTCAATGAAAGGGACACT-3′	HMB	0.6 ± 1.0	0.4 ± 0.3	0.6 ± 1.2	0.6 ± 1.1	0.5 ± 0.7	0.5 ± 0.7	0.4 ± 0.9	0.3 ± 0.4
**Calpain**									
5′-GCCAAGCAGGTGAAC-3′	PLA	1.0 ± 1.0	0.7 ± 0.4	0.7 ± 0.3	1.2 ± 1.1	0.4 ± 0.3	0.5 ± 0.3	0.5 ± 0.3	0.7 ± 0.5
5′-TGAAGTCTCGGAATGACATC-3′	HMB	0.45 ± 0.3	0.3 ± 0.1	0.4 ± 0.3	0.6 ± 0.4	0.5 ± 0.3	0.3 ± 0.1	0.4 ± 0.2	0.4 ± 0.2

*Significantly different from rest leg at each timepoint and within in each condition P < 0.05.

## 4 Discussion

In the present study, we sought to determine the effects of HMB-FA supplementation adjunct to RET on muscle function and mass in older aged men. Further using D_2_O, we aimed to make the first longer-term measures of the effect of HMB-FA on MPS. Our results show that gains in muscle function (i.e. 1RM, MVC) and muscle mass induced by 6 weeks of RET were largely similar between the groups. Nonetheless, HMB-FA supplementation exhibited a marginally more favourable response in relation to gains in thigh fat free mass over placebo, supported by an enhanced early increase in MPS over the first two weeks of RET.

The anabolic efficacy of HMB has already been trialed in many scenarios, including to enhance muscle mass and function in relation to athleticism, ageing, and cachectic diseases [11]. In the present study, we found that HMB-FA supplementation provided some incremental effect in relation to muscle hypertrophy as evidenced by increases in thigh fat free mass by DXA. However, we were unable to identify any differences when quantifying muscle thickness by ultrasound. Both DXA and ultrasound measures of muscle mass have inherent limitations; for instance, DXA tends to overestimate absolute skeletal muscle contractile mass [24]. Contrarily, ultrasound measures of muscle thickness tend to be made at a single point along the VL muscle, so it does not account for muscle cross sectional area, nor necessarily reflect the entire VL nor indeed the thigh muscles as a whole. From our data, we can only conclude that the positive influence of HMB-FA on thigh fat free mass by DXA reflects a small but generalized improvement in thigh muscle mass, not reflected by measuring VL muscle thickness alone. These data demonstrating improvements in muscle mass gains with HMB-FA vs. placebo are also in line with other studies [16], although unlike this previous study we did not see any changes in muscle mass of the non-exercising leg. We speculate that the greater duration of that study and greater subject numbers might explain this difference. Likewise, we have observed improvements in functional measures of muscle strength such as 1-RM and MVC after just 3 weeks of RET in both groups, which is in agreement with studies previously performed showing an increase in muscle strength coupled with muscle hypertrophy (Rabita et al., 2000; Seynnes et al., 2007), suggesting earlier adaptive responses to training than previously reported (Fatouros et al., 2006; Granacher et al., 2009). We observed an increase in 1-RM after 6 weeks of training in both groups, with similar improvements between the treatment and placebo groups by the end of study period; in contrast to some studies reporting a significant gain in strength and functionality with HMB supplementation compared to the placebo [29], [30]. However, it has to be taken into account that these studies were conducted over longer periods (8 and 12 weeks, respectively). Further, one study also included supplementation of lysine and arginine in addition to HMB [30]. It is possible that the rapid neural adaptations known to predominate during the early stages of a resistance training program could have masked any additional beneficial effect of HMB-FA on muscle strength during this relatively short training duration. In terms strength improvements assessed by MVC, there was an accelerated response in the HMB-FA group initially, as we observed significant improvements by 3 weeks of study period in the HMB-FA group only, although both the groups increased significantly in MVC strength by the end of the study.

A similar randomised double blind placebo controlled trial carried out in two phases previously reported that body composition, muscle function and strength in older individuals (>65, men and women) were enhanced by calcium HMB (Ca-HMB) supplementation without RET [16]. They conducted the study over a period of 24 weeks in two phases where placebo or 3 g/d Ca-HMB was given to older men and women either alone (Phase I) or in combination with RET (Phase II). The results of phase II of this study, which was similar to our study design where effects of Ca-HMB were seen in combination with RET, stated that total, arm and leg lean mass, muscle strength and function increased significantly in both RET groups however there no group differences, this is consistent with our findings; our study is novel in that we have also measured myofibrillar MPS in addition to the expression of a number of genes involved in muscle hypertrophy/atrophy and myogenic regulatory factors in response to each acute RET bout coupled with PLA vs HMB-FA supplementation.

The mechanisms by which HMB might impose efficacy include its known acute actions upon stimulating MPS and suppressing MPB [12]. To seek mechanistic explanations for any effects of RET/HMB interventions, we quantified MPS using D_2_O methodologies. Despite there being no treatment interaction between PLA and HMB-FA over the first 2 weeks of RET, MPS did show significant increases in the HMB-FA group only. We have previously shown that muscle adaptation is most active during the first 3 weeks of an RET program [4]; with MPS robustly increased during this active period of muscle growth, becoming attenuated thereafter despite progressive training [31]. Our inability to distinguish significant differences between HMB-FA vs. placebo groups at any given timepoint likely reflects the small incremental effects on muscle mass gains apparently induced by HMB-FA supplementation, an effect that may have become prevalent with a larger cohort as our study was primarily powered to investigate MPS increases. Nonetheless, we cannot conclude that a suppression of MPB might also be a more dominant mechanism of HMB's anabolic actions in relation to RET in humans. Long term measures of MPB are technically challenging, but “reverse labelling” D_2_O approaches may be feasible options to determine the impact of HMB (or other interventions) on both MPS/MPB in future.

With a view to seek potential gene level regulation of RET/HMB interventions, we also quantified the expression of a number of genes that have been associated with muscle anabolism (i.e. those that regulate myogenesis/ribosomal biogenesis) in response to acute bouts of RET. In doing so we found that acute RET had a minimal impact upon most of the genes we measured; this may be related to a blunted gene expression response to exercise in older age [32], although without a direct comparison to a younger group herein, this remains speculation. In contrast c-myc was upregulated consistently after bouts of RET and represented a robust “biomarker” of acute responses to RET; c-myc is an oncogene regulating cellular growth and has been long known to be regulated by muscle loading [33] and in this report was even shown to be blunted in an avian model of muscle stretch induced hypertrophy. Nonetheless, c-Myc was clearly and consistently upregulated in our group of older men, although this was unaffected by HMB-FA supplementation. As such, from the targeted gene data we have here, HMB-FA had no impact upon the expression of many key growth genes either in non-exercising or exercising human skeletal muscle. It is possible that, due to the timing of the muscle biopsy, we may have missed the acute upregulation of some of the genes we measured [34], [35], however, we saw no effect of chronic HMB-FA supplementation in the non-exercise limb over 6 weeks. It could be argued thus, that the major effects of HMB-FA are through post-translational effects on signalling pathways regulating MPS and MPB; many studies support this notion [36].

In conclusion, we have shown that HMB-FA supplements appear to induce a marginal improvement in muscle mass gains (via DXA), in line with previous studies in this area [16]. Nonetheless, this did not translate into functional improvements as assessed by isometric and isokinetic (1-RM) strength tests. MPS was significantly elevated during the first two weeks only in the HMB-FA supplemented group, returning to baseline levels during the last two weeks, similar to the response we observed previously in young healthy male volunteers undertaking an identical training regime [4]. Nonetheless, the present study adds to the current weight of literature that tends to favour efficacy of HMB and HMB containing supplements in ageing, in relation to skeletal muscle outcomes [37].

## Funding sources

This work was supported by the Medical Research Council Grant MR/K00414X/1 and Arthritis Research UK, (ARUK) Grant 19891 as part of the MRC-ARUK Centre for Musculoskeletal Ageing Research, The Dunhill Medical Trust Grant R364/1112 (to KS, PJA, DJW, MVN & JPW); and the Physiological Society (awarded to PJA & KS).

## Conflicts of interest

The authors declare no conflicts of interest. JAR is an employee of Metabolic Technologies Inc., who supplied the free acid-HMB on a collaborative basis and undertook the HMB plasma analyses, but were blinded to the sample identity. Metabolic Technologies Inc., has patents pending on HMB free acid, and market HMB to sports nutrition companies.

## Declaration of authorship

Study Design KS, PJA, DJW, JWW & MVN.

Conduct of Study UD, MB, AS, JQ, CB, HA, MF, BEP, JWW.

Laboratory Analyses & Functional Measures UD, MB, AS, JQ, MF, BEP, DJW, JAR.

Data Collection and Analysis UD, MB, AS, JQ, MF, BEP, DJW.

Interpretation and Writing Manuscript UD, MB, PJA, KS.

Read and approved Manuscript All authors.
